# Stem-like CD8^+^ T cells in cancer

**DOI:** 10.3389/fimmu.2024.1426418

**Published:** 2024-08-15

**Authors:** Chelsea Steiner, Nathan Denlinger, Xiaopei Huang, Yiping Yang

**Affiliations:** Division of Hematology, The Ohio State University Comprehensive Cancer Center, Columbus, OH, United States

**Keywords:** stem-like CD8 T cells (T_SL_), chronic viral infection, cancer models, immune, tertiary lymphoid structure (TLS), tumor microenvironment (TME)

## Abstract

Stem-like CD8^+^ T cells (T_SL_) are a subset of immune cells with superior persistence and antitumor immunity. They are TCF1^+^ PD-1^+^ and important for the expansion of tumor specific CD8^+^ T cells in response to checkpoint blockade immunotherapy. In acute infections, naïve CD8^+^ T cells differentiate into effector and memory CD8^+^ T cells; in cancer and chronic infections, persistent antigen stimulation can lead to T cell exhaustion. Recent studies have highlighted the dichotomy between late dysfunctional (or exhausted) T cells (T_LD_) that are TCF1^–^ PD-1^+^ and self-renewing TCF1^+^ PD-1^+^ T_SL_ from which they derive. TCF1^+^ T_SL_ cells are considered to have stem cell-like properties akin to memory T cell populations and can give rise to cytotoxic effector and transitory T cell phenotypes (T_TE_) which mediate tumor control. In this review, we will discuss recent advances made in research on the formation and expansion of T_SL_, as well as distinct niches required for their differentiation and maintenance in the setting of cancer. We will also discuss potential strategies to generate these cells, with clinical implications for stemness enhancement in vaccine design, immune checkpoint blockade (ICB), and adoptive T cell therapies.

## 1 Introduction

Immune checkpoint blockade (ICB) therapy has generated impressive success in recent years as 15~30% of cancer patients treated with ICB experience durable remissions (1). It has been proposed that ICB can reverse exhausted or late dysfunctional CD8^+^ T cells (T_LD_) to an effector-like state. However, recent studies have shown T_LD_ cells have a terminally differentiated phenotype and may not be readily rescued. Rather, proliferative bursts of a relatively undifferentiated population of “stem-like” T cells (T_SL_) occur after ICB, which has been correlated with clinical benefit. These T_SL_ are identified by their expression of transcription factor T cell factor-1 (TCF1), along with intermediate expression of inhibitory receptor, programmed cell death protein-1 (PD-1). TCF1^+^ PD-1^+^ T_SL_ cells have the ability to expand, self-renew, and differentiate into transitory effector-like T cells (T_TE_) and T_LD_ cells. T_SL_ cells have been identified to play a vital role in sustaining the CD8^+^ T cell response in both chronic infection and cancer. Their presence is associated with positive clinical outcomes of checkpoint immunotherapies in patients with melanoma, colorectal, and non-small cell lung cancer (NSCLC) (2–4). Here we will review the latest developments regarding T_SL_ population formation and expansion, along with the specific niches required for their maintenance and differentiation in the context of cancer. We will also explore potential approaches to produce T_SL_ cells and discuss the therapeutic implications of enhancing stemness in adoptive T cell therapies, ICB, and vaccine design.

## 2 Formation, expansion, and hallmarks of stem-like CD8^+^ T cells

**Figure 1:**
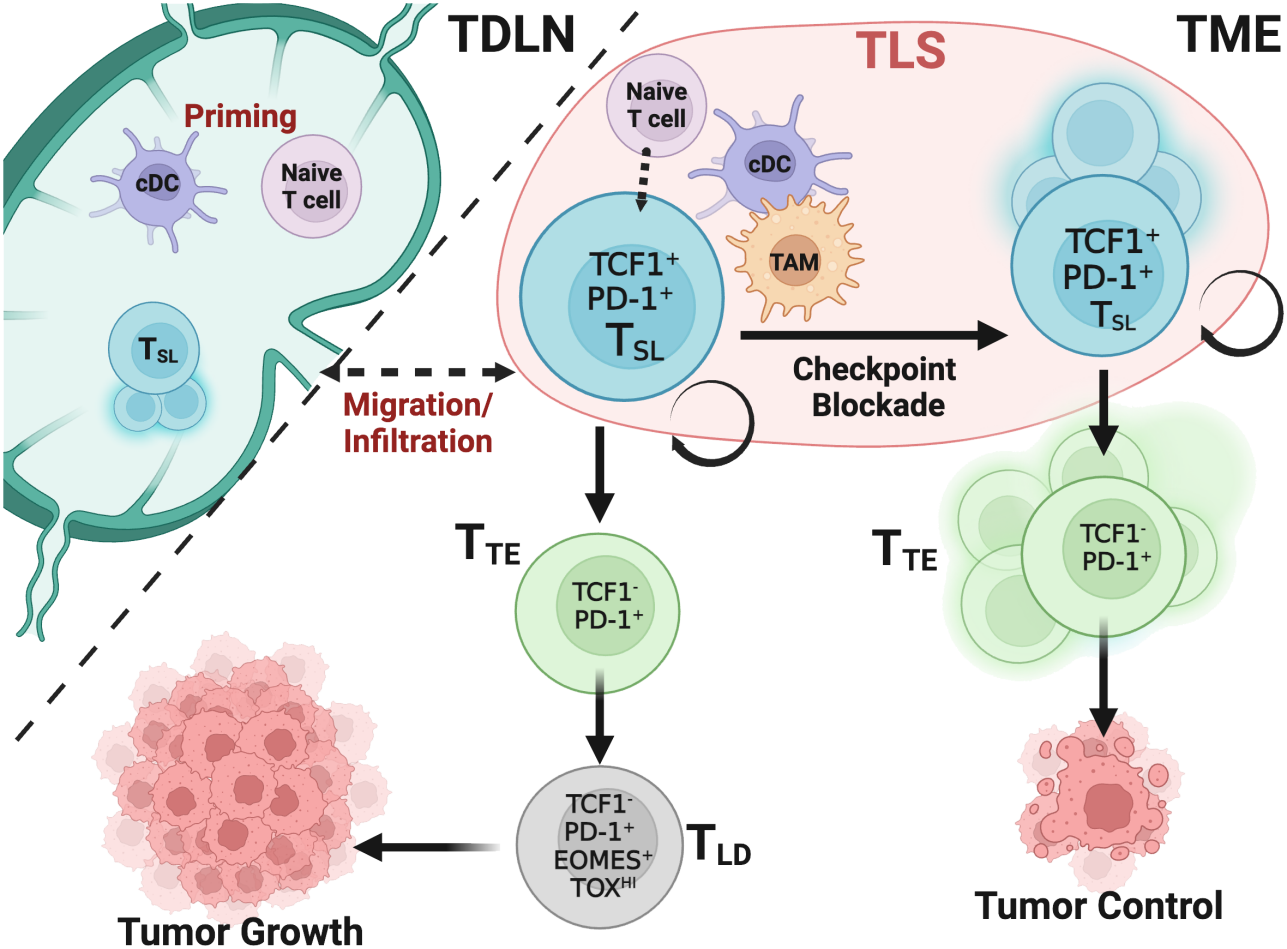
T_SL_ cells drive and maintain CD8^+^ T cell responses in cancer after ICB. Naïve and T_SL_ CD8^+^ T cells are primed and activated in the tumor draining lymph node (TDLN) or tertiary lymphoid structures (TLS) within the tumor by conventional dendritic cells (cDCs) that present tumor derived antigen. A portion of these activated T_SL_ cells reside in the TDLN and maintain a reservoir that migrate and infiltrate the tumor microenvironment (TME). Maintenance of T_SL_ cells has yet to be fully determined within these immunological niches. Without ICB, following activation, T_SL_ cells infiltrate tumors and rapidly undergo exhaustion in the presence of persistent antigen stimulation. While transitory effector CD8^+^ T cells (T_TE_) cells differentiate from T_SL_ cells, T_TE_ quickly adopt a late dysfunctional (T_LD_) phenotype but can carry a level of some tumor control through cytotoxic cytokines and tumor cell targeting. Upon ICB, the T_SL_ population undergoes self-renewal and proliferation, giving rise to the T_TE_ subset and this supports the majority of the CD8^+^ T cell antitumoral response, leading to tumor control.

**Figure 2:**
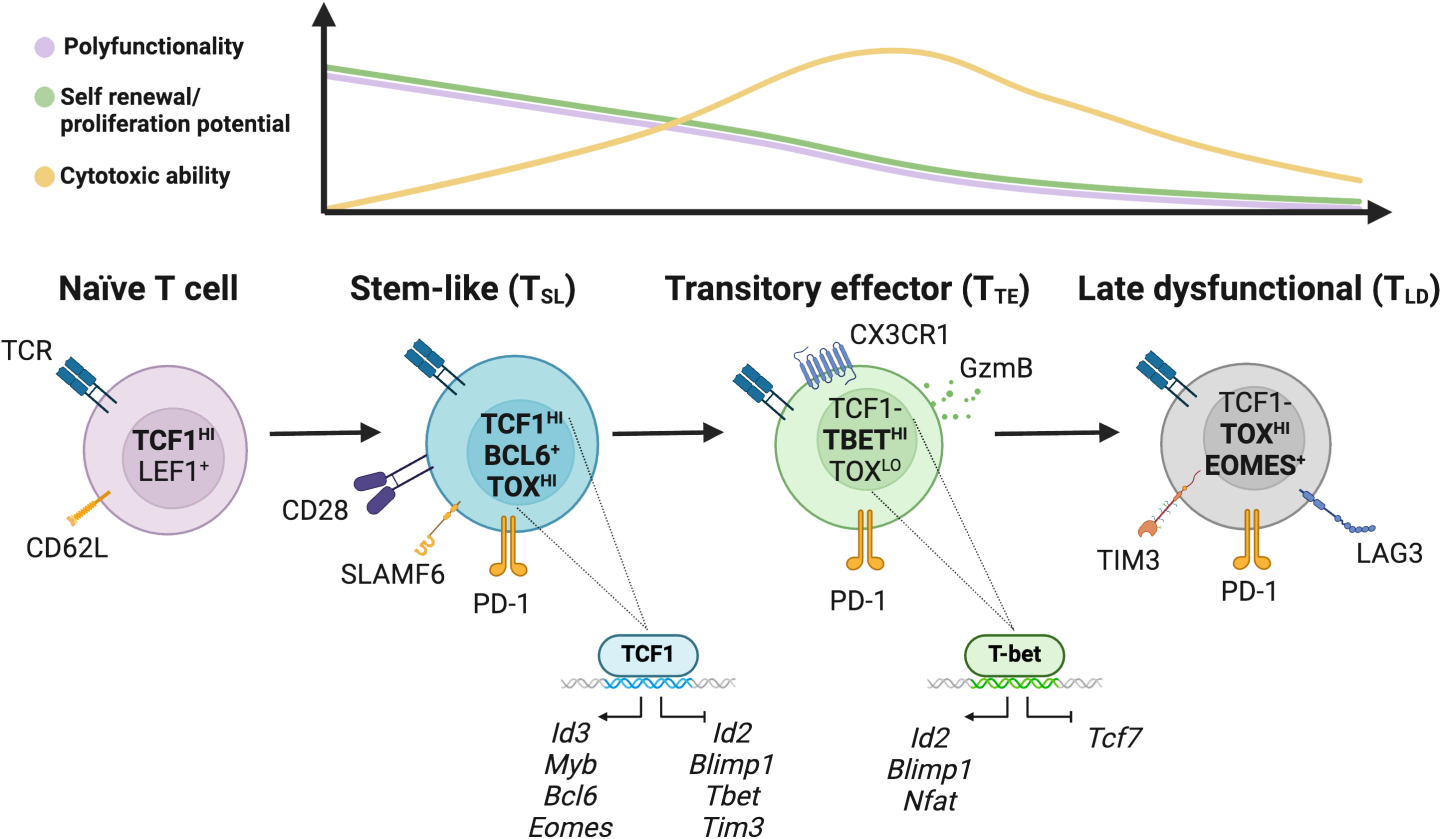
Model of the characteristics and differentiation of CD8^+^ T cell states in cancer. The transcription factors TCF1 and LEF1, as well as the adhesion and lymphocyte homing molecules CD62L, are highly expressed in naïve T cells. Downstream, the population of stem-like CD8^+^ T (T_SL_) cells with strong polyfunctionality and self-renewal ability is defined by TCF1. These cells have a strong proliferation capacity, are primarily quiescent *in vivo*, and are able to support the CD8^+^ T cell response. By suppressing the expression of effector-associated genes like Id2, Blimp-1, Tbet and Tbx21 and stimulating memory-associated genes like Eomes, Myb, Bcl-6, TCF1 facilitates the generation, maintenance, and functionality of these cells. Phenotypically, T_SL_ cells display CD28 and ICOS costimulatory markers, low or moderate amounts of PD-1, SLAMF6, and CXCR5. T_SL_ cells expand upon ICB and both maintain the T_SL_ reservoir and differentiate into further subpopulations. Differentiation of T_SL_ give rise to downstream transitory effector CD8^+^ T cells (T_TE_) that express high PD-1 receptor, proliferate rapidly in steady state down regulating TCF1 expression, and upregulate T-BET. T-BET inhibits TOX-mediated development of late dysfunctional T cells (T_LD_) phenotype in T_TE_ cells. Transitory cells exhibit the expression of CX3 chemokine receptor 1 (CX3CR1) and PD-1. T_TE_ proliferate to help target and eliminate tumor cells. After chronic antigen stimulation, these T_TE_ cells develop into T_LD_ cells which are characterized by high expression of checkpoint receptors (PD-1, TIM3, LAG3, CTLA-4, TIGIT, and CD101), poor polyfunctionality, low proliferation capacity, but retain some cytotoxic potential.

Stem-like CD8^+^ T cells have emerged as key players in response to ICB, as a subset of cells that retain stemness, have memory potential, and a high proliferative capacity. Targeting the PD-1: PD-L1 pathway with ICB treatment drives the expansion of these cells. This was first observed in chronic infection models (5–8) and subsequently in mouse and human cancers (2–4, 9, 10). As shown in **Figure 1**, the proliferation burst encompasses not only expansion of T_SL_’S cells’ downstream T_TE_ progeny, but also self-renewal of the T_SL_ population. T_SL_ self-propagate an epigenetically distinct, stable pool of T_SL_ cells that persists during active disease. This population is armed for subsequent proliferative bursts and fuels a downstream differentiated effector population in an antigen-dependent manner. T_SL_ cells survive and persist following antigen withdrawal, similar to conventional memory cells. Additionally, they can mount a recall response and continue to produce terminally differentiated progeny (11, 12). Although this subset is more proliferative than other differentiated exhausted subsets, compared to conventional memory cells, T_SL_ have reduced proliferative capacity and cytokine function (13). T_SL_ cells do share many markers with memory and naïve T cells (**Figure 2**; **Table 1**). Markers such as CD62L and CD27 are more commonly expressed on naïve and memory populations, while CCR7 and CD28 are often expressed by both naïve and T_SL_ cells. They are also induced/maintained by some similar transcription factors (TFs) including TCF1, BCL6, FOXO1, STAT3, JUN, MYB, BACH2, EOMES, TOX and ID3 (5–7, 14, 15). However, while T_SL_ cells share many memory and stem-like features, they are committed to the exhaustion lineage, and transfer an exhausted phenotype to their progeny (16). While ICB treatment results in the expansion or proliferative bursts of this stem-like population, these cells and their effector progeny show distinct epigenetic features and metabolic state of exhausted T cells (17–19). Studies have observed that although commitment toward the T cell exhaustion phenotype begins as early as 5 days, it requires time for the epigenetic imprint to stabilize where it cannot be overcome by ICB (16, 20–22). The TF nuclear factor of activated T cells (NFAT) plays a pivotal role in effector and exhaustion responses of CD8^+^ T cells and induces the effector program with its associate TF activator protein 1 (AP-1) and its subunits JUN/FOS (23). In the absence of AP-1, NFAT induces a program of negative feedback leading to T cell exhaustion. Downstream targets of NFAT: TOX, NR4A1, NR4A2 are critical in enforcing T cell exhaustion in T_SL_ cells (24–27). Absence of TOX results in the loss of the T_SL_ population and loss over time in their effector progeny in chronic infection and tumor models (5, 7, 25, 26). Likewise, a recent study reported double deletion of NR4A1/NR4A2 in CD8^+^ tumor-infiltrating lymphocytes (TILs) resulted in murine tumor eradication after transfer as well as expansion of T_SL_ population with increased chromatin accessibility of several stem-like/memory-related genes (28). T_SL_ cells, however, do not express other co-inhibitory, exhausted T cell markers (TIM3, TIGIT, CTLA4) but do express low to intermediate levels of PD-1, not as a marker of exhaustion but rather activation (29). PD-1 has been shown to help preserve the co-expressing PD-1^+^ TCF1^+^ T_SL_ population by attenuating TCR and co-stimulatory CD28, and by repressing downstream effector differentiation (22, 30, 31). T_SL_ also express other markers such as inducible T cell costimulator (ICOS) molecule, CD28, CXCR5, SLAMF6 (also known as LY108), which denote a population of cells that have experienced antigen and require lymphoid homing (3–6, 14, 32). In chronic viral infection, T_SL_ infiltrate B cell follicles correlating with CXCR5 expression on T_SL_ whereas in tumors, SLAMF6 is highly expressed and positively correlates with TCF1 levels (2, 4, 9, 33).

**Table 1:** Summary of the transcription factors, biomarkers, and key features that define CD8^+^ T cell subsets in cancer.

Murine■ Human■ Both■	Naïve T cell	T Stem-like (T_SL_)	Transitory Effector (T_TE_)	Late Dysfunctional (T_LD_)
**Transcription Factors**	TCF1, LEF1	TCF1, LEF1, EOMES, TOX, MYB, FOXO1, JUN, STAT3 ID3, BCL6, BACH2, EGR2	TBET, BLIMP1, BATF, IRF4, ID2, NFAT, RUNX3, NR4A	EOMES, TOX, BATF, NFAT
**Biomarkers**	CD62L CCR7 CD28 CD27 CD45RA CD45	TCF1 PD-1 LY108/SLAMF6 CXCR5 CD28 ICOS CCR7 CD69 CD45RO	PD-1 GZMB TBET LAG3 CX3CR1 CD45RO	PD-1 TIM3 LAG3 TIGIT CD101 CTLA4 CX3CR1 CD45RO
**Key Features**	Immature cell Circulate in lymph and blood Feeds downstream subsets	Self-renewal Expands/proliferate after ICB Persistent population pool Feeds downstream effector subsets	Effector/cytotoxic killing to control tumor growth	Increased expression of inhibitory receptors Limited killing capacity and proliferation

All figures created with BioRender.com.

Another critical feature of T_SL_ cells is the uniform expression of TCF1, encoded by the *Tcf7* gene, which is essential for the formation and function of this population (3, 5–7, 10, 14). Originally identified as a TF essential for thymocyte development, both TCF1 and its homologue LEF1, are now known to promote memory T cell differentiation and inhibit effector differentiation (34, 35). Open chromatin sites in T_SL_ cells are highly enriched in the TCF/LEF motif, similar to naïve T cells, and overlap frequently with TCF1 binding peaks, suggesting direct regulation by TCF1 (20, 35, 36). Studies in chronic infection and tumor models have shown that loss of TCF1 in CD8^+^ T cells limits their maintenance, function, and overall response to ICB, but does not diminish their overall function (3, 7, 10, 37). Additionally, a preclinical tumor study showing ectopic expression of TCF1 skews TILs to adopt a T_SL_ phenotype while enhancing their polyfunctionality and further suppressing inhibitory receptors and modulating the transcriptome to further suppress TFs like BLIMP1, RUNX3, and TOX to improve viral and tumor control (38). A recent study disputes that tumor immunogenicity dictates reliance on TCF1 for ICB efficacy (39). However, antitumor responses in poorly immunogenic tumors can be improved by optimizing T cell priming through either vaccination or enhancing antigen presentation on tumors (39). Additionally, frequency of *TCF7-*expressing CD8^+^ T cells in melanoma can correlate to positive response to ICB, whereas in advanced clear cell renal carcinoma patients, it failed to predict any clinical outcomes (40, 41). How TCF1 directly aids in forming and expanding this crucial stem-like population within its environment is still debated.

Together, the key features that define the formation and expansion of T_SL_ cells encompass multiple regulatory pathways. Many of the features of T_SL_ are similar to other well defined T cell subsets, therefore it is crucial to establish how regulatory mechanisms operate uniquely in the T_SL_ population in a variety of environments. We have described how T_SL_ cells self-renew while maintaining an exhausted lineage; next we will delve into how this subset continues to feed into the pool of CD8^+^ T cells and help sustain responses to ICB.

## 3 Differentiation and maintenance of stem-like CD8^+^ T cells

Studies from chronic viral infection and tumor models have characterized two populations of epigenetically and spatially distinct populations of CD8^+^ T cells: TCF1^+^ PD-1^+^ TIM3^-^ CD8^+^ T_SL_ and their progeny, TCF1^-^ PD-1^+^ CD8^+^ T transitory effector-like CD8^+^ T cells (T_TE_) (3–7, 9, 10). The T_TE_ cells become terminally differentiated, late dysfunctional TCF1^lo/-^ PD-1^+^ TIM3^+^ T cells (T_LD_) that carry distinct transcriptional and epigenetic programs that differ from those seen in traditional memory and effector populations, both in cancer and chronic viral infection (**Figures 1**, **2**; **Table 1**) (8, 19, 22). It has been shown that T_SL_ drive the proliferative response after immunotherapy and are often associated with clinical benefit, while T_LD_ populations have limited survival and re-expansion potential (3–7, 10, 42). T_SL_ cells and their progeny are committed to an exhausted phenotype, however a unique feature of the T_SL_ population being its ability to be stimulated to expand by ICB, whereas T_LD_ cells cannot be reinvigorated (5, 7, 8, 16, 43). On the other hand, the majority of the tumor specific population exhibits a T_LD_ phenotype, which may indicate a continuous immune response that requires a precursor population generating and infiltrating from external locations (37, 44–49).

The generation and maintenance of T_SL_ cells may be significantly impacted by varying environmental cues. In chronic infection, most T_SL_ cells are located within B cell follicles and the T cell zone of the spleen while their progeny exist within the red pulp taking up residency rather than migration (6, 14, 50). Contrastingly in tumors, T_SL_ cells migrate between perivascular niches or tertiary lymphoid structures (TLS) within the tumor and reservoirs in the tumor-draining lymph node (TDLN) (**Figure 1**) (3, 9, 32, 51–61). Blocking migration using sphingosine 1-phosphate receptor 1 (S1P1)-agonist FTY720 in multiple preclinical tumor models prevented tumor regression and challenged the understanding that anti-PD-1 immunotherapy primarily targets intratumoral T cells. This also suggests that T_SL_ migration to TDLN may even be required for T_SL_ cell maintenance (51, 52, 55, 60, 61). These specific tissue niches likely have two purposes for maintaining T_SL_ cells: to sequester away this population from inflammatory cues that quickly drive differentiation into exhausted phenotypes and to provide close, tightly regulated contact with antigen-presenting cells (APCs) such as dendritic cells (DCs) (60, 62). Recent preclinical research also implies that molecularly distinct lymph-node resident CD8^+^ memory-like and T_SL_ cells are sole mediators of ICB (61, 63). Two additional recent studies in non-small cell lung cancer (NSCLC) and head and neck squamous cell carcinoma (HNSCC) respectively, also support the idea that T_SL_ cells respond to immunotherapy within the lymph nodes (64, 65). Clusters of T_SL_ populations and APCs are also linked to significant T cell infiltration in human malignancies, whereas their absence may lead to immune evasion (9, 34). While T_SL_ cells are clustered with APCs and even CD4^+^ T cells within TLSs creating a supportive network to promote effective differentiation into T_TE_ subsets, T_LD_ cells are more scattered throughout the tumor parenchyma where they can readily engage with target cells (57, 66–68). Evidence also suggests that immunotherapy responses in sarcoma, melanoma and renal cell carcinoma are favorably linked with TLSs containing B cells and T_SL_ cells (33, 69, 70). In tumors that possess obstacles preventing the infiltration of T cells, such as solid tumors, immune cell niches can persist and harbor concentrated populations of T_SL_ cells that are aggregated with APCs (71). Cells in such niches were able to rapidly regenerate the immune response in patients with brain metastases and these immune niches were prognostic for local disease control (71). Thus, it is likely the interactions of T_SL_ cells with DCs and B cells within these niches are influential in the maintenance and function of T_SL_ and are required for durable CD8^+^ T cell responses. As previously mentioned, epigenetic analysis of T_SL_ in chronic infection compared to T_LD_ revealed unique open chromatin sites, and T_SL_ subsets show increased accessibility to XCL1 which is involved in the interactions between DCs and T cells (3, 4, 36, 72). XCL1 expressed on T cells promotes the recruitment of XCR1^+^ conventional type 1 DCs (cDC1) which have superior antigen processing and cross-presentation capabilities (73). Several groups have highlighted the necessity of cDC1s in sustaining T_SL_ cells and inducing the proliferative burst after ICB within the TLS as well as in maintaining the TDLN T_SL_ reservoir in preclinical tumor and chronic infection models, and patient samples (60, 62, 74). Additionally, the B7/CD28 pathway, expressed on DCs and T cells respectively, may have a role in structuring how these interactions sustain the immune response as T_SL_ have high CD28 expression that is necessary for the proliferative burst after ICB (75, 76). By blocking B7 costimulatory molecule on APCs or deletion of CD28 on T cells, effective responses to PD-1/PD-L1 ICB were diminished (76).

Another environmental cue being investigated is the CXCR3 pathway as a significant axis of immunotherapy response that regulates the infiltration and spatial positioning of T cells near APCs expressing the ligands CXCL9/10/11 within the murine and human tumor microenvironment (TME) (54, 77, 78). As multiple myeloid populations within the TME express the ligand CXCL9, including both DCs and tumor-associated macrophages (TAMs), and these chemokines are broadly induced in response to treatment, it remains another avenue to investigate in the maintenance of T_SL_ within TLSs (79–82). In the TME, macrophages are more abundant and express higher levels of CXCL9 than compared to DCs and may play a more prominent role in the TME compared to DCs in the TDLN (81).

Differentiation of T_SL_ into their cytolytic progeny T_TE_ cells has proved vital to the efficacy of ICB. The maintenance of this population via the TDLN reservoir or in TLSs within the tumor additionally have gained recognition in contributing to improved clinical outcomes. Many of the networks and signaling pathways involved in these environments will likely aid in determining future successes of therapeutics.

## 4 Therapeutic potential of stem-like CD8^+^ T cells in cancer

### 4.1 Immune checkpoint blockade

ICB therapy against inhibitory receptors PD-1 and CTLA4 of TILs has shown success in mounting a T cell response against tumors in many cancer types. Efficacy is highest in tumors with more mutational burden and typically higher TIL infiltration suggesting leveraging an already present immune response. Prior to the role of T_SL_, it was thought that T_LD_ being “rescued” from their late dysfunctional phenotype to a less exhausted, more effector phenotype was the primary mechanism of ICB (83). Some clinical studies have shown an abundance of cells with a T_TE_ or T_LD_ phenotype rather than T_SL_ cells can provide a better predictor for response to ICB (84–88). While TCF1^+^ expression by TILs in human melanoma coincides with clinical benefit of ICB, TCF1 is produced also by bystander TILs which are less relevant for antitumor response. High frequencies of TCF1^+^ PD-1^+^ T_SL_ thus may be an unreliable biomarker as a portion of these cells are not tumor-specific (40, 89). Likely, the ratio of T_SL_ to more differentiated TILs may represent a more suitable biomarker for outcome prediction as T_SL_ frequencies are comparable to those observed in responders versus non-responders (4, 40). In chronic infection and tumor models, T_SL_ have been shown to be critical in amplifying the response to ICB by self-renewal, expansion, and differentiation into T_TE_, supplying the pool of cytotoxic cells and mediating disease control (90). Given their crucial role in ICB, it is imperative to effectively control T_SL_ cells. Continuous driving of differentiation by immune checkpoints can negatively impact maintenance of T_SL_ cells and ultimately result in loss of the ability to expand and differentiate, driving patients toward a refractory state (22, 91, 92). Bi-specific antibody therapy has shown promising outcomes in patients with hematologic malignancies, although in cancers more resistant to ICB and favorable outcomes are limited. One drug construct uses an anti-PD-1 molecule as a targeting moiety fused to a stimulatory IL-2 variant (IL-2v) to deliver IL-2 to PD-1^+^ T cells in the TME. Combining with anti-PD-L1 treatment resulted in murine tumor regression, enhanced infiltration of the T_SL_ population, and reprogramming of TAMs (93). It is important to note, prolonged exposure of T cells to bispecifics through continuous infusion can also cause cells to adopt the T_LD_ phenotype and therefore must be carefully evaluated (93, 94). Other therapeutic strategies taken to clinical trial include inhibiting cell division, T cell receptor (TCR) signaling, or epigenetic pathways to hinder T_SL_ differentiation (18, 19, 95–97). Additionally, depleting or altering T cell signaling pathways in T_SL_ cells have shown to promote stem-like phenotype retention, allowing these cells to persist in harsh environments that would otherwise push these populations towards T_LD_ phenotype, and instead still produce effective T_TE_ progeny (98–100). Clinical data has also shown that ICB therapy induced expansion of pre-treatment T_SL_ cells present in patients who were responders compared to non-responders which had more pre-treatment T_LD_ phenotypes, experienced therapy resistance (10, 40, 48, 49, 59, 90).

Quantity or presence of T_SL_ alone may be insufficient as a marker of response, because as previously mentioned, APC-dense niches or TLSs tolerant for T_SL_ self-renewal or expansion, may additionally be required for effective responses. Clinical observations have revealed that tumors with such regions correlate with favorable therapeutic responses (51, 59, 71, 87, 88). Additionally in other preclinical studies it has been observed that blocking T cell egress from TDLN, surgically removing the TDLN, or disrupting the migration of T cells from the TME diminishes ICB response (51–54, 61). Further new studies from patient samples of NSCLC, HNSCC, and melanoma also indicate T_SL_ cells respond to ICB directly in the TDLN, displaying local clonal expansion and subsequent migration of these new clones to the TME (44, 64, 65, 83, 90). Therefore, targeting the establishment and cultivation of these regions within the TME or TDLN, to enhance T_SL_ maintenance and differentiation, could further increase efficacy (101, 102).

### 4.2 Adoptive cellular therapy

This therapy encompasses two main approaches: ex vivo expansion of TILs or genetic modification of peripheral blood mononuclear cells (PBMC)-derived T cells for tumor specific subsets and subsequent reintroduction into the patient. Ex vivo manufacturing and expansion strategies to induce T_SL_ cells include introducing IL-7, IL-15, and IL-21 to promote expression of associated genes like *TCF7*, *Eomes*, and *Bcl6* (103–107), or promoting Notch signaling upstream of TCF1 (108, 109). Suppressing genes associated with late dysfunctional or exhaustive phenotypes such as Tbet, BATF, EOMES pharmacologically ex vivo can maintain stem-like genes (TCF1/LEF1) and retains T_SL_ cell polyfunctionality (110, 111). Numerous studies of both preclinical models and patients of ACT observe that less differentiated, memory and stem-like cells elicit more of an effective antitumoral response (112–118). Genetically engineering T cells using retroviral transduction to incorporate a tumor reactive TCR or a chimeric antigen receptor (CAR-T) has become standard of care for many hematologic malignancies (119–125). Increased populations of terminally exhausted CD8^+^ CAR-T cells present in pre-treatment product correlate with worse outcomes however, presence of more naïve and memory-like CAR-T phenotypes are correlated with increased response rates (126–128). Although extensive clinical research into T_SL_ phenotypes in CAR-T products has yet to be conducted, one recent study identified that PD-1^+^ TCF1^+^ stem-like CAR-T and PD-1^+^ TIM3^+^ effector-like CAR-T correlated with improved clinical outcomes (129). This study highlights the importance of PD-1 expression on CAR-T cells post-infusion as a marker of activation rather than exhaustion for optimal activation as well as the potential for optimizing stem-like phenotypes in CAR-T subsets to potentially improve clinical outcomes.

Study of the epigenetic landscape of T_SL_, T_TE_ and T_LD_ subsets has revealed several targets for controlling the differentiation and antitumor response and are now in preclinical CAR-T models (130–133). Exploration of the chromatin accessibility of CAR-T cells at the single cell level, both *in vitro* and *in vivo*, identified two distinct subsets (133). The subsets consisted of intermediate exhausted CAR-T cells enriched for TFs of T_SL_ cells (JUN/FOS) and another with enriched motifs of BATF and IRF4 resembling terminally exhausted or the T_LD_ CAR-T subset. CAR-T cells with knockdown of BATF, IRF4 or NR4A expression had enhanced effector function, inhibited exhaustion and prolonged CAR-T cell persistence *in vivo* (133, 134). A dual knockout of genes *PRDM1* (encoding BLIMP1 TF) and *NR43A* in preclinical murine models, skewed CAR-T cell phenotypes toward T_SL_ subsets and away from T_LD_, improving antitumor responses and not achieved by single knockouts (132).

Additionally, several preclinical CAR-T models targeting overexpression of TFs specific for T_SL_ such as c-Jun and FOXO1, promote stem-like phenotypes, enhanced expansion potential, persistence and therapeutic efficacy *in vivo* (130, 131). Other factors such as hub transcription factors, like FOXP1 and KLF2 that have high numbers of enhancers that are positioned in the center of gene regulatory networks, can serve as checkpoints that control lineage-defining TFs between stem-like and effector CAR-T, and the decision between effector and late dysfunctional CAR-T cells, respectively (135). While harnessing the power of T_SL_ cell phenotype in CAR-T therapy by targeting key transcriptional regulators may lead to further successful trials, investigating the relationships of other immune cells or combination therapy in altering other environmental cues could be crucial to their advancement.

Pre-existing TLS or APC-dense niches may also be required for generating stem-like CAR-T phenotypes and catering to the cultivation of these environments may also increase their persistence (63, 136, 137). Utilizing stem-like CD8^+^ T cells and their respective molecular determinants as biomarkers of response to CAR-T may also prove beneficial within a clinical setting.

Cancer immunotherapy such as ICB and CAR-T rely on T cell infiltration. The accumulated evidence above shows that combining multiple therapeutic agents is crucial for cancer immunotherapy and targeting stem-like CD8^+^ T cells requires more than one approach.

### 4.3 Cancer vaccination

Studies in the forefront of cancer vaccination are seeking to harness the self-renewal, long lasting durability, and sustainability of the T_SL_ subset by targeting common tumor antigens or patient specific neoantigens (neoAg) (138–140). Recent advances in genomic sequencing have led to personalized cancer vaccines targeting neoAg. Early studies show feasibility in mice and clinical trials, but neoAg targeted CD8^+^ T cell responses have been limited (139–145). Coupling self-assembling nanoparticle vaccine platform technology, exploiting its ability to quickly drain via lymphatics to DCs and enhance antigen presentation to CD8s, the SNP-7/8a intravenous vaccination generated more T_SL_ cells that are receptive to ICB in a therapeutic murine model (146). Additionally, adenovirus (Ad)-vectored vaccines encoding tumor neoAg combined with ICB have been shown to eradicate large tumors and increases in T_SL_ cells in the TDLN and T_TE_ cells within the TME in mice and have translated into similar results within the clinic (147). Further, studies harnessing not only T_SL_ cells but also other tumor targeting progenitors, like stem-like natural killer (NK) cells are gaining interest. Introduced at the contraction phase after immunization with an artificial adjuvant vector cell (aAVC), an IL-2/anti-IL-2 monoclonal antibody complex (IL-2Cx) combination activated stem-like subsets that correlated with therapeutic responses, and induced long-term memory CD8^+^ T cells that conferred protection against tumor rechallenge in a leukemic model (148). While tumor vaccine trial successes have been mixed, expanding the population of tumor specific T_SL_ cells is likely the key consideration for the future of favorable tumor vaccine outcomes.

## 5 Conclusions and outlook

The role of stem-like T cells has been underscored in recent studies, highlighting their potential to improve the antitumor effect of immunotherapies. However, to fully exploit this potential, a complete understanding of how T_SL_ cells form, maintain, and function is necessary. Recent advances in deciphering this subsets’ key characteristics and hallmarks have led even further to questions that require investigation. The most vital questions and potential targets will likely center around T_SL_ and APC interactions within their relevant niches in a variety of models. The targeting and harnessing of T_SL_ cells will require multiple points of application.

In conclusion, while significant strides have been made in understanding the role and potential of T_SL_ cells in cancer therapy, there is still much work to be done. Future research should focus on elucidating the regulatory circuits that control these cells, understanding the APC interactions with intratumoral T_SL_ cells and within niches, and developing methods for T_SL_ cell generation. These efforts will be crucial in harnessing T_SL_ cells for therapeutic interventions and enhancing immunotherapy against cancer. The exploration of combination therapies and strategies to maintain the “stemness” of T cells represent promising avenues for future research and could revolutionize cancer treatment.
